# Sequences in overpartitions

**DOI:** 10.1007/s11139-022-00685-y

**Published:** 2023-01-17

**Authors:** George E. Andrews, Ali K. Uncu

**Affiliations:** 1 Department of Mathematics, The Pennsylvania State University, University Park, PA 16802 USA; 2 Johann Radon Institute for Computational and Applied Mathematics, Austrian Academy of Science, Altenbergerstrasse 69, 4040 Linz, Austria; 3 Department of Computer Science, Faculty of Science, University of Bath, Bath, BA2 7AY UK

**Keywords:** Overpartitions, Partition identities, Determinants, Primary 11B65, Secondary 11C08, 11C20, 11P81, 11P84, 05A10, 05A15, 05A17

## Abstract

This paper is devoted to the study of sequences in overpartitions and their relation to 2-color partitions. An extensive study of a general class of double series is required to achieve these ends.

## Introduction

The topic of sequences in partitions goes back to the work of Sylvester [15] and MacMahon [14]. In 2004, Holroyd, Liggett and Romik found the concept central to a problem in bootstrap percolation [11]. Subsequently there have been a number of papers on this topic by Andrews [3, 4], Bringmann et al. [7], Choi et al. [8]. The topic was also considered in the unpublished Ph.D. thesis of Hirschhorn [10] and by many others.

This paper has its genesis in two identities discovered empirically by Ali K. Uncu.

### Theorem 1.1

1.1 $$\begin{aligned} \sum _{m,n\ge 0} \frac{(-1)^n q^{\frac{3n(3n+1)}{2} + m^2+ 3mn}}{(q;q)_m(q^3;q^3)_n} = \frac{1}{(q;q^3)_\infty }. \end{aligned}$$

The second identity is still unproven.

### Conjecture 1.2

1.2 $$\begin{aligned} \sum _{m,n\ge 0} \frac{(-1)^n q^{\frac{3n(3n+1)}{2} + m^2+ 3mn +m + n}}{(q;q)_m(q^3;q^3)_n} = \frac{1}{(q^2,q^3;q^6)_\infty }, \end{aligned}$$

where

$$\begin{aligned}(A;q)_n := \prod _{j=0}^{n-1} (1-Aq^j),\text { and }(A_1,A_2,\dots ,A_r;q) := \prod _{i=1}^r(A_i;q)_n. \end{aligned}$$

In light of the fact that the double series in (1.1) and (1.2) are clearly cousins of many of the generating functions alluded to in the first paragraph, it is not surprising that these series are special cases of more general series related to the theory of partitions.

In this paper, we shall study the following series and their applications to overpartitions:

1.3 $$\begin{aligned} F(i,k;x) = \sum _{m,n\ge 0} \frac{(-1)^n q^{{(2k+1)n+1\atopwithdelims ()2} + m^2 + (2k+1)mn + i(m+n)} x^{m+(2k+1)n}}{(q;q)_m (q^{2k+1};q^{2k+1})_n}. \end{aligned}$$

We note that *F*(0, 1; 1) is the series in Theorem 1.1 and *F*(1, 1; 1) is the series in the Conjecture 1.2.

Indeed, our focus will, for the most part, be on the polynomial refinements of (1.1) and (1.2). Namely, for $k,j,N\ge 0$,

$$\begin{aligned}&F_N(i,j,k;x,q)\\&\quad =F_N(i,j,k;x)=F_N(i,j,k) \\&\quad =\left\{ \begin{array}{ll} \begin{array}{l}\displaystyle \sum _{m,n\ge 0} (-1)^n q^{{(2k+1)n+1\atopwithdelims ()2} + m^2 + (2k+1)mn + i(m+n)} x^{m+(2k+1)n} \\ \displaystyle \quad \times {N-(2k+1)n-m+j\brack m}_q {N-2kn -m \brack n}_{q^{2k+1}}\end{array},&{} \text {if }N\ge 0, \\ \\ 0, &{} \text {if }N<0,\\ \end{array} \right. \end{aligned}$$

where

$$\begin{aligned} {A\brack B}_q := \left\{ \begin{array}{ll}0 &{} \text {if } B>A\text { or }B<0, \\ \dfrac{(q;q)_{A}}{(q;q)_B(q;q)_{A-B}},&{}\text {otherwise.} \end{array} \right. \end{aligned}$$

Note $\lim _{N\rightarrow \infty } F_N(0,j,1;1)$ is the series in the Theorem 1.1 and $\lim _{N\rightarrow \infty } F_N(1,j,1;1)$ is the series in the Conjecture 1.2.

As we will show in Sect. 5

$$\begin{aligned}\lim _{N\rightarrow \infty } \frac{F_N(i,0,k;1)}{(q;q)_\infty } \end{aligned}$$

is the generating function for a general class of overpartitions.

It should be noted that Theorem 1.1 has the following direct consequence which will also be proved in Sect. 5.

### Corollary 1.3

The number of overpartitions of *N*, where for any $k\ge 1$

i. $\overline{k}+\overline{(k+1)}$ does not appear, and
ii. there are no sequences of the form $\overline{1}+2+\overline{3}+4+\overline{5}+\dots +(2k)+\overline{(2k+1)}$,

equals the number of partitions of *N* into red and green parts with each green part $\equiv 1$ (mod 3).

For example, when $N=4$, the 13 overpartitions in the first class are

$$4,\ \overline{4},\ 3+1,\ \overline{3}+1,\ 3+\overline{1},\ \overline{3}+\overline{1},\ 2+2,\ \overline{2}+2,\ 2+1+1,\ $$

$$ \overline{2}+1+1,\ 2+\overline{1}+1,\ 1+1+1+1,\ \overline{1}+1+1+1,$$

and the 13-colored partitions in the second class are

$$ 4_r,\ 4_g,\ 3_r+1_r,\ 3_r+1_g,\ 2_r+2_r,\ 2_r+1_r+1_r,\ 1_r+1_g+1_r,\ $$

$$ 2_r+1_g+1_g ,\ 1_r+1_r+1_r+1_r,\ 1_g+1_r+1_r+1_r,\ $$

$$1_g+1_g+1_r+1_r,\ 1_g+1_g+1_g+1_r,\ 1_g+1_g+1_g+1_g.$$

Section 2 will be devoted to determining the recurrences and *q*-difference equations satisfied by $F_N(i,j,k;x)$ and Sect. 3 will consider identities arising when $k=0$.

Section 4 will provide the proof of Theorem 1.1. Section 5 will consider overpartition applications. Section 6 is devoted to some continued fraction expansions related to Theorem 1.1 and their implications.

## Recurrence and *q*-difference equations for $F_N(i,k;x)$

In the following, we shall use the standard notations,

$$\begin{aligned} (A;q)_n:= & {} \prod _{j=0}^{n-1}(1-Aq^i),(A;q)_\infty :=\prod _{j=0}^{\infty }(1-Aq^i),\\ \end{aligned}$$

and

$$\begin{aligned} {A \brack B}_q&:= \left\{ \begin{array}{ll}0 &{} \text {if } B>A\text { or }B<0, \\ \dfrac{(q;q)_{A}}{(q;q)_B(q;q)_{A-B}},&{}\text {otherwise.} \end{array} \right. \end{aligned}$$

### Theorem 2.1

2.1 $$\begin{aligned} F_N(i,j,k;x)= & {} F_{N-1}(i,j,k;x)+ x q^{N+j+i-1} F_{N-2}(i,j,k;x) \nonumber \\{} & {} - x^{2k+1} q^{(2k+1)(N-k)+i} F_{N-(2k+1)}(i,j,k;x).\nonumber \\ \end{aligned}$$

### Proof

$$\begin{aligned}&F_N(i,j,k;x) - F_{N-1}(i,j,k;x) - x q^{N+j+i-1} F_{N-2}(i,j,k;x) \\&\qquad + x^{2k+1} q^{(2k+1)(N-k)+i} F_{N-(2k+1)}(i,j,k;x)\\&\quad = \sum _{m,n\ge 0} (-1)^n q^{{(2k+1)n+1\atopwithdelims ()2} + m^2 + (2k+1)mn + i(m+n)} x^{m+(2k+1)n} \\&\qquad \times \left\{ {N-(2k+1)n-m+j\brack m}_q {N-2kn-m\brack n }_{q^{2k+1}}\right. \\&\qquad -{N-1-(2k+1)n-m+j\brack m}_q{N-1-2kn-m\brack n}_{q^{2k+1}}\\&\qquad -q^{N-(2k+1)n-2m+j} {N-1-(2k+1)n-m+j\brack m-1}_q {N-2kn -m-1\brack n}_{q^{2k+1}}\\&\qquad \left. -q^{(2k+1)(N-(2k+1)n-m)} {N-(2k+1)n-m+j\brack m}_q {N-2kn -m-1\brack n-1}_{q^{2k+1}} \right\} \end{aligned}$$

Let us combine the first and the last term in the braces in (2.2). This yields

2.2 $$\begin{aligned}&{N-(2k+1)n-m+j \brack m}_q \left\{ {N-2kn-m \brack n}_{q^{2k+1}} \right. \nonumber \\&\qquad \left. - q^{(2k+1)(N-(2k+1)n-m)}{N-2kn-m-1\brack n-1}_{q^{2k+1}} \right\} \nonumber \\&\quad ={N-(2k+1)n-m+j \brack m}_q{N-2kn-m-1\brack n}_{q^{2k+1}} \end{aligned}$$

by [2, (3,3,3), p.35].

Next we combine the second and the third terms in the braces of (2.2). This yields

2.3 $$\begin{aligned}&-{N-2kn-m-1 \brack n}_{q^{2k+1}} \left\{ {N-(2k+1)n-m+j-1 \brack m}_q\right. \nonumber \\&\qquad \left. + q^{N-(2k+1)n-2m+j}{N-(2k+1)n-m+j-1\brack m-1}_q \right\} \nonumber \\&\quad =-{N-(2k+1)n-m+j\brack m}_q{N-2kn-m-1 \brack n}_{q^{2k+1}} \end{aligned}$$

by [2, (3,3,3), p.35].

Thus we see that combining the second and the third term (2.3) is the negative of the term we get by combining the first and the last terms (2.2) of (2.2). So the expression inside the braces of (2.2) vanishes and the theorem is proven. $\square$

### Corollary 2.2

For $$N\ge 1$$,

2.4 $$\begin{aligned} F_N(0,1,1;x) = (1+xq^N) F_{N-1}(0,1,1;x) -x^2q^{2N-1} F_{N-2} (0,1,1;x). \end{aligned}$$

### Proof

First, we note the initial conditions

$$\begin{aligned} F_{-1}(0,1,1;x)&=0,\\ F_0(0,1,1;x)&=1,\\ F_1(0,1,1;x)&=1+xq,\\ F_2(0,1,1;x)&=1+(q + q^2)x, \end{aligned}$$

and

$$\begin{aligned} F_3(0,1,1;x)&=1+(q+q^2+q^3)x+x^2 q^4-x^3 q^6. \end{aligned}$$

Thus we check directly that (2.4) is true for $1\le N\le 3$.

If $N>3$, we know from Theorem 2.1 that

$$\begin{aligned} 0&= F_N(0,1,1;x)-F_{N-1}(0,1,1;x) -xq^N F_{N-2}(0,1,1;x)\\&\quad + x^3 q^{3N-3} F_{N-3}(0,1,1;x)\\&=(F_N(0,1,1;x) - (1+xq^N) F_{N-1}(0,1,1;x) +x^2q^{2N-1} F_{N-2} (0,1,1;x))\\&\quad + xq^N (F_{N-1}(0,1,1;x) - (1+xq^{N-1}) F_{N-2}(0,1,1;x)\\&\quad +x^2q^{2N-3} F_{N-3} (0,1,1;x)). \end{aligned}$$

Let

$$\begin{aligned}S(N) = F_N(0,1,1;x)- (1+xq^N) F_{N-1}(0,1,1;x) +x^2q^{2N-1} F_{N-2} (0,1,1;x), \end{aligned}$$

then we have just shown that

2.5 $$\begin{aligned} S(N) + xq^N S(N-1)=0.\end{aligned}$$

But we began with the observation that for $1\le N\le 3$, $S(N)=0$. Hence, (2.5) and these initial conditions prove that $S(N)\equiv 0$ for all $N\ge 1$. $\square$

### Theorem 2.3

2.6 $$\begin{aligned} F_N(i,j,k;x) = F_N(i,j-1,k;x) + x q^{N+i+j-1} F_{N-1} (i,j-1,k;x). \end{aligned}$$

### Proof

$$\begin{aligned}&F_{N}(i,j,k;x)-F_N(i,j-1,k;x)\\&= \sum _{m,n\ge 0} q^{ {(2k+1)n+1\atopwithdelims ()2} +m^2 +(2k+1)mn + i(m+n) } x^{m+(2k+1)n} {N-2kn-m\brack n}_{q^{2k+1}}\\&\quad \times \left\{ {N-(2k+1)n-m+j\brack m}_q - {N-(2k+1)n-m+j-1\brack m}_q\right\} \end{aligned}$$

by [2, (3,3,3), p.35]

$$\begin{aligned}&= \sum _{m,n\ge 0} q^{ {(2k+1)n+1\atopwithdelims ()2} +m^2 +(2k+1)mn + i(m+n) } x^{m+(2k+1)n} {N-2kn-m\brack n}_{q^{2k+1}}\\&\quad \times \left\{ q^{N-(2k+1)n-2m+j} { N-(2k+1)n-m+j-1 \brack m-1}_q\right\} \\ \end{aligned}$$

Now, by shifting the summation variable $m\mapsto m+1$ and rewriting the terms, we get

$$\begin{aligned}&= \sum _{m,n\ge 0} q^{ {(2k+1)n+1\atopwithdelims ()2} +(m+1)^2 +(2k+1)(m+1)n + i(m+1+n) } x^{m+1+(2k+1)n}\\&\quad \times {N-2kn-m-1\brack n}_{q^{2k+1}}\\&\quad \times \left\{ q^{N-(2k+1)n-2(m+1)+j} { N-(2k+1)n-m+j-2 \brack m-1}_q\right\} \\&= x q^{N+i+j-1} F_{N-1}(i,j-1,k;x). \end{aligned}$$

$$\square $$

### Theorem 2.4

2.7 $$\begin{aligned}{} & {} F_N(i,0,k;x) - F_{N-1}(i,0,k;xq) - xq F_{N-2}(i,0,k;xq^2) \nonumber \\{} & {} \quad + x^{2k+1}q^{{2k+2\atopwithdelims ()2}+i} F_{N-(2k+1)}(i,0,k;xq^{2k+1})=0. \end{aligned}$$

### Proof

This result follows easily from the determinant representation of $F_N(i,0,k;x)$:

$$\begin{aligned}&F_N(i,0,k;x)\\&= {\left| \begin{array}{cccccccccc} 1 &{} xq^{1+i} &{} 0 &{} 0 &{} \dots &{} 0 &{} -x^{2k+1} q^{(2k+2)\cdot 1+i} &{} 0 &{}\dots &{} 0 \\ -1 &{} 1 &{} x q^{2+i} &{} 0 &{} \dots &{} &{} 0&{}-x^{2k+1} q^{(2k+2)\cdot 2+i}&{} &{} \vdots \\ 0 &{} -1 &{} 1 &{} xq^{3+i} &{}\ddots &{} &{} &{} &{} &{} \\ 0 &{} 0 &{} -1 &{}\ddots &{} \ddots &{} &{} &{} &{} &{} \\ \vdots &{} \vdots &{} &{} \ddots &{} &{} &{} &{} &{} &{}\vdots \\ &{} &{} &{} &{} &{} &{} &{} &{} \ddots &{} 0\\ &{} &{} &{} &{} &{} &{} &{} &{} &{} -x^{2k+1} q^{(2k+1)(N-k)+i}\\ &{} &{} &{} &{} &{} &{} &{} &{} \ddots &{} 0\\ &{} &{} &{} &{} &{} &{} &{} &{} \ddots &{} \vdots \\ &{} &{} &{} &{} &{} &{}\ddots &{} \ddots &{} \ddots &{} 0\\ &{} &{} &{} &{} &{} &{} \dots &{} 1 &{} x q^{N-2+i} &{} 0 \\ \vdots &{} &{} &{} &{} &{} &{} \dots &{} -1 &{} 1 &{} x q^{N-1+i}\\ 0 &{} \dots &{} &{} &{} &{} &{}\dots &{} 0 &{} -1 &{} 1\\ \end{array}\right| } \end{aligned}$$

Expansion along the last column reveals that this expression satisfies Theorem 2.1, and the expansion along the top row proves the assertion of this theorem. $\square$

We remark that the recursion of Theorem 2.4 is restricted to $j=0$ is because for $j>0$, the initial values of the determinant do not match the corresponding values of $F_N(i,j,k;x)$.

### Corollary 2.5

2.8 $$\begin{aligned} F_N(0,1,1;x) = (1+ xq)F_{N-1}(0,1,1;xq) - x^2 q^3 F_{N-2}(0,1,1;xq^2).\end{aligned}$$

### Proof

We deduce from Corollary 2.2 that

$$\begin{aligned} F_{N}(0,1,1;x):= \left| \begin{array}{cccccccccc} 1+xq &{} -x^2q^3 &{}0 &{} \dots &{} &{} 0 \\ -1 &{} 1+ x q^2 &{} -x^2q^5 &{} 0 &{} \dots &{} &{} &{} &{} \vdots \\ 0 &{} -1 &{} 1+xq^3 &{} \ddots &{} &{} &{} &{} &{} \\ \vdots &{} 0 &{} -1 &{} \ddots &{} &{} &{} &{} &{} \\ &{} &{} \vdots &{} \ddots &{} &{} &{} &{} &{} \\ &{} &{} &{} &{} &{} &{} &{} &{} \vdots \\ &{} &{} &{} &{} &{} &{} \ddots &{} \ddots &{} 0 \\ \vdots &{} &{} &{} &{} \ddots &{} 0 &{} -1 &{} 1+ xq^{N-1} &{} -x^2q^{2N-1} \\ 0 &{} \dots &{} &{} &{} &{} \dots &{} 0 &{} -1 &{} 1+xq^N \end{array} \right| \end{aligned}$$

Expanding along the last column reveals that this expression is indeed $F_N(0,1,1;x)$ by Corollary 2.2. Expansion along the top row yields (2.8). $\square$

## Reduction for $k=0$

### Theorem 3.1

3.1 $$\begin{aligned} F_N(i,j,0;x) = \sum _{n=0}^{j-1} {j-1\brack n }_q x^n q^{n(N+i+1)}. \end{aligned}$$

### Proof

$$\begin{aligned} F_N(i,j,0;x)&= \sum _{m,n\ge 0}x^{m+n} (-1)^n q^{{n+1\atopwithdelims ()2}+m^2 +mn+i(m+n)}\\&\quad \times {N-n-m+j\brack m}_q {N-m\brack n}_q \end{aligned}$$

Letting $M=n+m$ we rewrite the right side expression as follows.

$$\begin{aligned}&= \sum _{M\ge 0}x^M (-1)^M q^{{M+1\atopwithdelims ()2} + iM} \sum _{m\ge 0 } (-1)^m q^{m\atopwithdelims ()2} {N-M+j \brack m}_q {N-m \brack M-m}_q \end{aligned}$$

We can use the *q*-Chu–Vandermonde summation formula [2, p.37, (3.3.10)] to simplify the inner sum and finish the proof.

$$\begin{aligned}&=\sum _{M\ge 0}x^M (-1)^M q^{{M+1\atopwithdelims ()2} + iM} {N\brack M}_q \frac{(-1)^M q^{NM-{M\atopwithdelims ()2}}(q;q)_{j-1}(q;q)_{N-M}}{(q;q)_{j-M-1}(q;q)_{N}}\\ {}&= \sum _{M\ge 0}x^M q^{M(N+i+1)} {j-1\brack M}_q. \end{aligned}$$

$$\square $$

## Proof of Theorem 1.1

### Theorem 4.1

For non-negative integers *N*,  let

4.1 $$\begin{aligned} f_N(q) := \sum _{j\ge 0} q^{3j^2 -2j} {N \brack 3j}_q (q^2,q^3)_j, \end{aligned}$$

then

4.2 $$\begin{aligned} f_{N+1}(q) = F_N(0,1,1;1) - q^N F_{N-1}(0,1,1;1). \end{aligned}$$

### Proof

The *q*-Zeilberger algorithm (implemented in [13] and many other places) is enough to prove that $f_N(q)$ satisfies the recurrence relation

4.3 $$\begin{aligned} (1 - q^{N-2}) f_{ N}(q)- (1 - q^{ 2 N-3}) f_{ N-1}(q) +q^{2 N-4} (1 - q^{ N-1}) f_{N-2}(q) =0.\nonumber \\ \end{aligned}$$

Corollary 2.2 shows that

$$\begin{aligned} F_{N}(0,1,1;1) -(1+q^{N})F_{N-1}(0,1,1;1)+q^{2N-1}F_{N-2}(0,1,1;1)=0 \end{aligned}$$

and similarly we can show that

$$\begin{aligned}\hat{F}_{N}(0,1,1;1) -q(1+q^{N-1})\hat{F}_{N-1}(0,1,1;1)+q^{2N-1}\hat{F}_{N-2}(0,1,1;1)=0, \end{aligned}$$

where $\hat{F}_N(0,1,1;1) = q^N F_{N-1}(0,1,1;1)$. We can find a recurrence satisfied by the sequence defined as the difference of $F_N(0,1,1;1)$ and $q^N F_{N-1}(0,1,1;1)$ using the closure properties of holonomic functions. These formal calculations are also included in the qGeneratingFunctions Mathematica package of Kauers and Koutschan [12]. Using the above recurrences we can then show that $b_{N+1} = F_N(0,1,1;1) - q^N F_{N-1}(0,1,1;1)$ satisfies the recurrence

4.4 $$\begin{aligned}&b_{N+1} -(1 + q) (1 + q^{N-1})b_{N} \nonumber \\&\quad +q\left( 1 +q^{2 N-4} + q^{2N-3} + q^{2N-2} + q^{N-1}+ q^{N-2}\right) b_{N-1}\nonumber \\&\quad - q^{2N-3} (1 + q) (1+ q^{N-2}) b_{N-2} +q^{4N-8} b_{N-3}=0. \end{aligned}$$

At this stage, to prove the lemma, one can either find a recurrence satisfied by the difference $f_N(q)$ and $b_N$ using the same approach or checks to see if the recurrences (4.3) and (4.4) have a common factor. The qFunctions package of Ablinger and Uncu has the implementation of finding a greatest common factor of two given recurrences. Using that we show that $f_N(q)$ and $b_N=F_{N-1}(0,1,1;1) - q^{N-1} F_{N-2}(0,1,1;1)$ satisfies the same second-order recurrence (4.3).

Now that we showed the left- and right-hand sides of (4.2) satisfy the same recurrence (4.3). All we need to do is to show that the initial values match. For $N=0$ and 1, it is easy to see that both sides of the claim gives 1. Therefore, since both sides satisfy the same second order recurrence with two equal initial conditions, the equation (4.2) is true for all $N\ge 2$. $\square$

Now we are finally equipped to prove Theorem 1.1. For $|q|<1$, taking the limit $N\rightarrow \infty$ of (4.2) we get

4.5 $$\begin{aligned} \sum _{j\ge 0} \frac{(q^2;q^3)_\infty }{(q;q)_{3j}} q^{3j^2-2j} = \sum _{m,n\ge 0} \frac{(-1)^n q^{\frac{3n(3n+1)}{2} + m^2+ 3mn}}{(q)_m(q^3;q^3)_n}. \end{aligned}$$

The right-hand side of (4.5) is the left-hand side of (1.1). Moreover, the left-hand side series in (4.5) can be summed using the *q*-Gauss sum [9, II.8, p.354],

$$\begin{aligned} \sum _{j\ge 0} \frac{(a,b;q)_j}{(q,c;q)_j} \left( \frac{c}{ab}\right) ^j = \frac{\left( \frac{c}{a},\frac{c}{b};q \right) _\infty }{\left( c,\frac{c}{ab};q \right) _\infty } , \end{aligned}$$

with $(a,b,c,q)\mapsto (\rho ,\rho ,q,q^3)$ and later $\rho \rightarrow \infty$. This finishes the proof of Theorem 1.1.

## Overpartitions and Proof of Corollary 1.3

An overpartition of *n* is an integer partition in which the first appearance of any summand may be overlined. For example, the eight overpartitions of 3 are

$$3,\ \overline{3},\ 2+1,\ 2+\overline{1},\ \overline{2}+1,\ \overline{2}+\overline{1},\ 1+1+1,\ \overline{1}+1+1.$$

In the following, we shall discuss sequences in overpartitions. We shall say that there is a sequence of the form $a_1 + a_2 +\dots + a_r$ in the given overpartition if for some $j\ge 0$, $(a_1+j) + (a_2+j) + \dots +(a_r+j)$ appears as a subpartition of the given partition. For example, there is a sequence of the form $\overline{1}+ 2+ \overline{3}$ in $4+\overline{5}+6+\overline{7}+7$ because $\overline{(1+4)} + (2+4)+ \overline{(3+4)}$ appears in the given overpartition.

### Theorem 5.1

$$\begin{aligned} \frac{F(0,0,k;x)}{(xq;q)_\infty } \end{aligned}$$

is the generating function for the overpartitions, where the exponent of *x* keeps track of the number of parts, in which

i. $\overline{j}+\overline{(j+1)}$ does not appear,
ii. there are no sequences of the form $\overline{1}+2+\overline{3}+4+\overline{5}+\dots +(2k)+\overline{(2k+1)}$.

If $$i>0$$,

$$\begin{aligned} \frac{F(i,0,k;x)}{(xq;q)_\infty } \end{aligned}$$

is the generating function for the overpartitions,in which

i. $\overline{j}+\overline{(j+1)}$ does not appear,
ii. the smallest overlined part is $$> i $$,
iii. sequences of the form
  $$ 2+3+\dots +i+\overline{(i+1)}+(i+1)+(i+2)+\overline{(i+3)} +(i+4) +\overline{(i+5)}+\dots +\overline{(2k)}+(2k+1) $$
  if *i* is odd, and
  $$2+3+\dots +i+\overline{(i+1)}+(i+1)+(i+2)+\overline{(i+3)} +(i+4) +\overline{(i+5)}+\dots +(2k)+\overline{(2k+1)} $$
  if *i* is even, are excluded.

For example, with $k=i=1$, the coefficient of $q^7$ in $F(1,0,1;x)/(xq;q)_\infty$ is

$$x^7 + 2x^6 + 4x^5 + 7x^4 + 10x^3 + 9x^2 + 2x.$$

The ten indicated partitions of 7 with 3 parts are

$$\begin{aligned}&5+1+1,\ \overline{5}+1+1,\ 4+2+1,\ \overline{4}+2+1,\ 4+\overline{2}+1,\ \overline{4}+\overline{2}+1,\ 3+3+1,\\&\quad \overline{3}+3+1,\ 3+2+2,\ \overline{3}+3+2. \end{aligned}$$

Note that $\overline{3}+\overline{2}+2$, $5+\overline{1}+1$, $3+\overline{2}+2$ have been excluded by the conditions *iii*., *iv*. and *v*., respectively.

### Proof

We take the limit as $N\rightarrow \infty$ in Theorem 2.4. Thus if

$$\begin{aligned} f(i,k;x) := \frac{F(i,0,k;x)}{(xq;q)_\infty }, \end{aligned}$$

then

5.1 $$\begin{aligned}&f(i,k;x,q):=f(i,k;x) = \frac{1}{(1-xq)}f(i,k;xq) + \frac{xq^{i+1}}{(1-xq)(1-xq^2)}f(i,k;xq^2)\nonumber \\&\quad -\frac{x^{2k+1}q^{{2k+2\atopwithdelims ()2}+i}}{(xq;q)_{2k+1}}f(i,k;xq^{2k+1}). \end{aligned}$$

Quite clearly *f*(*i*, *k*; *x*) is uniquely defined by (5.1) given the boundary conditions $f(i,k;x,0) = f(i,k;0,q)=1$. Hence to prove our theorem, we need only to show that the generating functions for the overpartitions in question satisfies the functional equation (5.1) plus the initial conditions. The boundary conditions are immediate from the fact that the empty partition of 0 is the only partition of non-positive number and the only partition with a non-positive number of parts.

Let us now examine the three components of the right-hand side of (5.1). The first term

$$\begin{aligned} \frac{f(i,k;xq)}{(1-xq)} \end{aligned}$$

clearly accounts for those overpartitions in question that do not have $\overline{i+1}$ as a part. The second term

$$\begin{aligned} \frac{xq^{i+1}}{(1-xq)(1-xq^2)}f(i,k;xq^2) \end{aligned}$$

accounts for those overpartitions where now $\overline{(i+1)}$ appear.

This term has introduced disallowed partitions. Particularly, it has juxtaposed $\overline{(i+1)}$ with the subpartition $2+3+\dots +i+\overline{(i+1)}+(i+1)+(i+2)+\overline{(i+3)} +(i+4) +\overline{(i+5)}+\dots +\overline{(2k)+(2k+1)}$ (an overline appears either on (2*k*) or on $(2k+1)$ as appropriate). This is not admissible.

However, the term

$$\begin{aligned} -\frac{x^{2k+1}q^{2+3+\dots +i+\overline{(i+1)}+(i+1)+(i+2)+\overline{(i+3)} +(i+4) +\overline{(i+5)}+\dots +\overline{(2k)+(2k+1)}}}{(xq;q)_{2k+1}}f(i,k;xq^{2k+1}) \end{aligned}$$

removes the offending overpartitions. Thus the right-hand side of (5.1) accounts for precisely those overpartitions described by Theorem 5.1. $\square$

We now prove Corollary 1.3.

### Proof of Corollary 1.3

We recall Theorem 1.1, which asserts

$$F(0,1;1) = \frac{1}{(q;q^3)_\infty }.$$

Hence,

5.2 $$\begin{aligned} \frac{F(0,1;1)}{(q;q)_\infty } = \frac{1}{(q;q)_\infty (q;q^3)_\infty }. \end{aligned}$$

The left-hand side of (5.2) is the generating function for the overpartitions described in Corollary 1.3 in the light of Theorem 5.1. The right-hand side of the generating function for colored partitions describe in Corollary 1.3. $\square$

## Some related continued fraction identities

One can easily see that the three term relations of Corollaries 2.2 and 2.5 give rise to finite continued fractions. We would like to start by recalling the initial conditions for $F_N(0,1,1;x)$ presented in the proof of Corollary 2.2:

$$\begin{aligned} F_0(0,1,1;x)=1\text { and }F_1(0,1,1;x)=1+xq. \end{aligned}$$

By a simple rearrangement of terms in Corollaries 2.2 and 2.5 and iteration of the formulas gives the two following results, respectively.

### Corollary 6.1

For $$N\ge 1$$,

6.1 $$\begin{aligned} \frac{F_N(0,1,1;x)}{F_{N-1}(0,1,1;x)} = 1+x q^N - \frac{x^2 q^{N-1}}{1+x q^{N-1} - \frac{x^2 q^{N-2}}{\ddots - \frac{\ddots }{1+x q^2 - \frac{x^2q}{1+xq}}}}. \end{aligned}$$

### Corollary 6.2

For $$N\ge 1$$,

6.2 $$\begin{aligned} \frac{F_N(0,1,1;x)}{F_{N-1}(0,1,1;x q)} = 1+x q - \frac{x^2 q^{3}}{1+x q^{2} - \frac{x^2 q^{5}}{\ddots - \frac{\ddots }{1+x q^{N-1} - \frac{x^2q^{2N-1}}{1+xq^N}}}}. \end{aligned}$$

The continued fraction (6.1) tends to 1 as $N\rightarrow \infty$. On the other hand, as $N\rightarrow \infty$, we get the following result from Corollary 6.2 at $x=1$ by employing Theorem 1.1 and simple manmipulations.

### Theorem 6.3

$$\begin{aligned} (q;q^3)_\infty \sum _{m,n\ge 0} \frac{(-1)^n q^{\frac{3n(3n+1)}{2} + m^2+ 3mn +m +3n+1}}{(q)_m(q^3;q^3)_n} = \frac{q}{1+ q - \frac{ q^{3}}{1+ q^{2} - \frac{ q^{5}}{1+q^3 - \frac{q^7}{\ddots }}}}. \end{aligned}$$

The right-hand side of Theorem 6.3 is closely related to a continued fraction noted by Ramanujan [5, 6]:

6.3 $$\begin{aligned} \frac{(q^2;q^3)_\infty }{(q;q^3)_\infty }=\frac{1}{1-\frac{q}{1+ q - \frac{ q^{3}}{1+ q^{2} - \frac{ q^{5}}{1+q^3 - \frac{q^7}{\ddots }}}}}. \end{aligned}$$

This yields the following theorem subject to some simple manipulations.

### Theorem 6.4

$$\begin{aligned} \sum _{m,n\ge 0} \frac{(-1)^n q^{\frac{3n(3n+1)}{2} + m^2+ 3mn +m +3n+1}}{(q;q)_m(q^3;q^3)_n} = \frac{1}{(q;q^3)_\infty }-\frac{1}{(q^2;q^3)_\infty }. \end{aligned}$$

Moreover, using (1.1) in Theorem 6.4, we get a series expansion for $1/(q^2;q^3)_\infty$ similar to (1.1).

### Theorem 6.5

$$\begin{aligned} \sum _{m,n\ge 0} \frac{(-1)^n q^{\frac{3n(3n+1)}{2} + m^2+ 3mn }(1-q^{m +3n+1})}{(q;q)_m(q^3;q^3)_n} = \frac{1}{(q^2;q^3)_\infty }. \end{aligned}$$

## Data Availability

Data sharing not applicable to this article as no datasets were generated or analysed during the current study.

## References

[1] Ablinger J, Uncu AK (2021). qFunctions—a mathematica package for $$q$$-series and partition theory applications. J. Symb. Comput..

[2] Andrews, G.E.: The Theory of Partitions, Cambridge Mathematical Library, Cambridge University Press, Cambridge (1998). Reprint of the original. MR1634067 (99c:11126) (1976)

[3] Andrews GE (2005). Partitions with short sequences and mock theta functions. Proc. Nat. Acad. Sci..

[4] Andrews, G.E.: Sequences in Partitions, Double $$q$$-Series, and the Mock Theta Functions $$\rho _3(q)$$, from Algorithmic Combinatorics-Enumerative Combinatorics, Special Functions and Computer Algebra, pp. 25–46. Springer, Cham (2020)

[5] Andrews, G.E., Berndt, B.C., Jacobsen, L., Lampede, R.L.: The continued fractions found in the unorganized portions of Ramanujan’s notebooks. Mem. Am. Math. Soc. 99(477) (1992)

[6] Andrews, G.E., Berndt, B.C., Sohn, J., Yee, A.J., Zaharescu, A.: On Ramanujan’s continued fraction for $$(q^2;q^3)_\infty /(q;q^3)_\infty $$. Trans. Am. Math. Soc. 355(6):2397–2411 (2003)

[7] Bringmann, K., Mahlburg, K., Nataraj, K.: Distinct parts partitions without sequences. Elec. J. Comb. 22 (2015)

[8] Choi Y, Kim B, Lovejoy J (2017). Overpartitions into distinct parts without short sequences. J. Num. Theo..

[9] Gasper G , Rahman M (2004). Basic Hypergeometric Series.

[10] Hirschhorn, M.D.: Developments in the theory of partitions, Ph.D. Thesis. University of New South Wales (1980)

[11] Holroyd AE, Liggett TM, Romik D (2004). Integrals, partitions, and cellular automata. Trans. Am. Math. Soc..

[12] Kauers, M., Koutschan, C.: A mathematica package for q-holonomic sequences and power series. Ramanujan J 19(2), 137–150 (2009)

[13] Koutschan, C.: Advanced Applications of the Holonomic Systems Approach, RISC, Johannes Kepler University, Linz. PhD Thesis. September (2009)

[14] MacMahon, P.A.: Combinatory Analysis, vol. 2. Cambridge University Press, Cambridge (1918) (Reprinted: Chelsea/AMS, Providence, 2001)

[15] Sylvester, J.J.: A constructive theory of partitions, arranged in three acts, an interaction and exodition. Am. J. Math. 5 (1882) pp. 251–330 and 6 (184), pp. 334–336 (1882–1884) (or pp. 1–83 of The Collected Mathematical Papers of J. J. Sylvester, Vol. 4. 12; reprinted by Chelsea, New York, 1974)

